# Secretome From Human Micro‐Fragmented Adipose Tissue Affects In Vitro Monocytes/Macrophages Inflammatory Activity by ICAM‐1 Expression

**DOI:** 10.1155/mi/9475320

**Published:** 2026-03-16

**Authors:** Valentina Coccè, Eleonora Martegani, Francesca Paino, Luisa Doneda, Giulio Alessandri, Barbara Manfredi, Aldo Giannì, Emilio Ciusani, Elena Colombani, Carlo Tremolada, Augusto Pessina

**Affiliations:** ^1^ Department of Biomedical, Surgical and Dental Sciences - CRC StaMeTec, University of Milan, Milan, Italy, unimi.it; ^2^ Department of Psychology, Catholic University of the Sacred Heart, Milan, Italy, unicatt.it; ^3^ Laboratory of Cellular Biochemistry and Molecular Biology, CRIBENS, Catholic University of the Sacred Heart, Milan, Italy, unicatt.it; ^4^ Maxillo-Facial and Dental Unit, Fondazione Ca’ Granda IRCCS Ospedale Maggiore Policlinico, Milan, Italy; ^5^ Department of Diagnostics and Technology, Fondazione IRCCS Istituto Neurologico “C.Besta”, Milan, Italy, istituto-besta.it; ^6^ Image Regenerative Clinic, Milan, Italy

**Keywords:** ICAM, inflammation, MCP-1, micro-fragmented adipose tissue, RANTES, secretome

## Abstract

Micro‐fragmented adipose tissue (MFAT) is regarded as one of the simplest and most practical biological preparations for clinical applications in tissue regenerative medicine. The clinical effectiveness of MFAT is attributed to its content of cells and growth factors that facilitate tissue regeneration. In this study, we investigated the biological activity of the secretome derived from cultured MFAT. Our primary focus was on its ability to influence the production of two inflammatory cytokines, RANTES (Regulated and Normal T Cell Expressed and Secreted) and MCP‐1 (Monocyte Chemoattractant Protein‐1), using ELISA assays, as well as its impact on the expression of Cell Adhesion Molecules (CAMs) on U‐937 macrophages via flow cytometry. We also explored the potential of the MFAT secretome to affect the proliferation of both normal and cancer cells. Our results showed that the MFAT secretome inhibited the production of MCP‐1 and RANTES, significantly reduced the expression of ICAM‐1 (Intercellular Adhesion Molecule 1) on U‐937 macrophages, and had no impact on the proliferation of normal or cancer cells. These findings suggest that the MFAT secretome is relatively safe and exhibits anti‐inflammatory properties, supporting the idea that its clinical effectiveness in treating joint inflammation may, in part, be due to its paracrine effects.

## 1. Introduction

Micro‐fragmented adipose tissue (MFAT) is a biological product derived from lipoaspirates (LAs) obtained through liposuction. After undergoing mechanical micro‐fragmentation and filtration, it results in a substance with a chemical composition similar to that of LA but with a more homogeneous structure, making it easier to handle. MFAT consists of cellular components like adipocytes, endothelial cells, mesenchymal stem/stromal cells, fibroblasts, and macrophages, with minimal blood cell contamination. It also contains acellular components such as the extracellular matrix, cytokines, enzymes, DNA, RNA, extracellular vesicles, and more [1]. MFAT can be produced using various techniques and medical devices that ensure minimal manipulation and no enzyme use. The Lipogems device, which gradually reduces adipose clusters, results in a particularly fluid form of MFAT and is already used in clinical settings for its regenerative and anti‐inflammatory benefits [2–4]. Currently, it is also used in autologous therapy for treating a range of conditions, including plastic/reconstructive surgery, osteoarthritis, and chronic ulcers (such as those caused by diabetes and Crohn’s disease) [5–8]. The clinical effectiveness of MFAT is largely attributed to the presence of growth factors such as G‐CSF (granulocyte colony‐stimulating factor), SCGF‐β (stem cell growth factor), and HGF (hepatocyte growth factor), which play a role in tissue regeneration and wound healing. Additionally, MFAT contains factors that stabilize the endothelial cells in blood vessels, potentially reducing inflammation‐induced activation of these cells [9].

The aim of this study was to further investigate whether the secretome derived from MFAT contains factors that influence macrophage activity in tissue inflammation and immune cell migration. To achieve this, we used an in vitro model of the human U‐937 cell line, which is widely regarded as an excellent model for studying inflammatory mechanisms. These cells exhibit monocyte/macrophage‐like properties and respond to inflammatory stimuli such as lipopolysaccharide (LPS) by producing key inflammatory mediators [10]. Our focus was on examining the modulation of two potent inflammatory cytokines, MCP‐1 (Monocyte Chemoattractant Protein‐1) [11] and RANTES (Regulated upon Activation, Normal T cell Expressed, and Secreted), both of which play essential roles in inflammation by recruiting cells to sites of inflammation [12]. The secretion of RANTES and MCP‐1 by U‐937 cells in response to MFAT secretome stimulation provided confirmation of the inflammatory or anti‐inflammatory effects of MFAT, as these chemokines are also involved “in vivo” in recruiting immune cells, such as T cells and monocytes, to sites of MFAT injection in clinical settings. Additionally, we examined the impact of the MFAT secretome on the expression of cell adhesion molecules (CAMs) in U‐937 cells, as these molecules are known to play a critical role during inflammatory responses [13, 14]. Finally, we assessed the biological activity of the MFAT secretome by evaluating its potential to influence the proliferation of various normal cell lines, including mesenchymal stromal cells, human skin fibroblasts (hSDFs), and keratinocytes, as well as human cancer cell lines, such as pancreatic carcinoma and melanoma. This investigation aimed to determine whether the secretome could affect cell proliferation, an important safety consideration for evaluating the clinical safety of MFAT.

## 2. Materials and Methods

### 2.1. Cell Lines

AT‐MSCs (adipose tissue‐derived mesenchymal stromal cells) were isolated from human adipose tissue and expanded in our laboratory as previously described [15]. The cells were used at passage number 3 and cultured in Dulbecco’s Modified Eagle Medium (DMEM) with low glucose (Euroclone, Pero, Milan, Italy), supplemented with 5% platelet lysate Stemulate (Cook Regentec, Indianapolis, IN, USA) and 2 mM L‐glutamine (Euroclone, Pero, Milan, Italy). The cells were incubated at 37°C in a 5% CO_2_ atmosphere. The expression of typical mesenchymal stem cell markers (CD90, CD105, and CD73), the absence of hematopoietic/endothelial markers (CD34, CD45, and CD31), and the ability to differentiate into mesodermal lineages (osteogenic, adipogenic, and chondrogenic) were confirmed as previously described [16]. Primary hSDFs (HuDe/BSPRC41), provided by Centro Substrati Cellulari, ISZLER (Brescia, Italy), and human keratinocytes (HaCaT, RRID: CVCL_0038) [17] were cultured in DMEM with low glucose, supplemented with 10% fetal bovine serum (FBS) (Euroclone, Pero, Milan, Italy) [18]. Several human cancer cell lines were also used, including human pancreatic adenocarcinoma cells (CFPAC‐1, RRID: CVCL_1119) [19], human melanoma (A375, RRID: CVCL_0132) [20], and human melanoma (M20, RRID: CVCL_DI51) [21]. These cells were maintained through weekly 1:10 passages in Iscove Modified Dulbecco’s Medium (IMDM) and 10% FBS. Additionally, the U‐937 cell line (RRID: CVCL_0007) was used in this study as a model for human monocyte‐like cells. All reagents were supplied by Euroclone (Pero, Milan, Italy). All cell lines tested negative for Mycoplasma.

### 2.2. Collection of LA and Microfragmentation

LA samples were obtained through liposuction of subcutaneous tissue using disposable cannulas provided with the Lipogems kit (Lipogems International, Milan, Italy). Following the acquisition of signed informed consent from the patient in accordance with the Declaration of Helsinki, tissue samples were collected from six women undergoing esthetic surgery, aged between 35 and 68 years (mean age: 54.16 ± 11.8 years). Approval for the use of these samples was granted by the Institutional Ethical Committee of Milan University (n. 58/23, C.E.UNIMI, 25.05.23).

MFAT specimens were prepared from the LA as previously described [3]. In brief, 50–100 mL of collected LA was transferred into a standard 225 mL device, where it was filtered for initial cluster reduction. The five stainless steel marbles inside the device were then shaken to disaggregate the fat material, producing cell clusters and micro‐fragmented fat tissue that migrated to the top of the device. Blood‐contaminating cells and unwanted fat residues were removed via a gravity counterflow of saline solution. When the solution inside the device appeared yellow and clear, the device was turned upside down. A second micro‐fragmentation was performed by using a syringe to push the adipose clusters through a size‐reduction filter. Upon completion, the MFAT product was aspirated into a syringe connected to the device and was ready for investigation.

### 2.3. Preparation of MFAT Secretome

Fresh MFAT (10 mL) from six donors (NS120422, BL060422, AF150422, BO310523, SF5523, and DFRP290523) was incubated with 30 mL of DMEM LG + 1% L‐glutamine (in the absence of FBS) in a 75 cm^2^ flask for 3 days at 37°C in 5% CO_2_. After 3 days, an additional 20 mL of DMEM LG was added, and the incubation continued for 2–3 more days. The entire volume was collected, placed in a conical tube, and centrifuged at 2500 g for 15 min. The floating MFAT was discarded, and the conditioned medium containing the secretome was collected and stored at −20°C until the freeze‐drying procedure.

### 2.4. Expression of Adhesion Molecules

U‐937 cells (5 × 10^6^) in a volume of 1 mL were stimulated with 1 mL of secretomes obtained from adipose tissue of various donors containing the same amount of proteins (36,8 + −4,2 μg/mL) evaluated by BCA assay (Thermofisher, USA): Negative control tests were conducted without any stimulation, whereas positive control tests involved stimulating U‐937 cells with LPS at a concentration of 1 μg/mL for 24 h, with the cells maintained at 37°C in 5% CO_2_. The expression of six adhesion molecules—VCAM (CD106), NCAM (CD56), ITGB3 (CD61), ICAM‐1 (CD54), MCAM (CD146), and ICAM‐2 (CD102)—was evaluated by flow cytometry using specific conjugated antibodies: VCAM‐1/PE (Beckman Coulter, cat. numebr B61547 AC), NCAM/APC‐750 (Beckman Coulter, cat. number B61871 AC), β3‐integrin/PC7 (Beckman Coulter, cat. number B62339 AC), ICAM‐1/PE (Caltag Laboratories, cat. number 322708), ICAM‐2/APC (Miltenyi Biotec, cat. number 130114305), and MCAM/FITC (BD Biosciences, cat. number 560846). Briefly, stimulated cells were collected, washed with PBS, and resuspended at a concentration of ~5·10^6^ cells/mL. One million cells from each treatment group were labeled with 1 μg of each antibody combination: VCAM/PE‐CD56(NCAM)/APC‐750/β3‐integrin/PC7 and ICAM‐1/PE‐ICAM‐2/APC‐MCAM/FITC. An appropriate combination of isotype antibodies was used to measure autofluorescence. Samples were incubated at 4°C for 1 h in the dark, and cells were fixed with 50 μL 1× fixative solution (Beckman Coulter). At least 10,000 events were acquired using a Navios EX flow cytometer (Beckman Coulter, IN, USA) and analyzed with Navios Platform System software (Beckman Coulter) [22].

### 2.5. Anti‐Inflammatory Activity

The anti‐inflammatory effect of the secretome was evaluated on U‐937 monocytes by measuring its capacity to inhibit or stimulate the production of two important inflammatory chemokines, RANTES and MCP‐1. The amount of secreted chemokines was determined using an ELISA assay following the manufacturer’s instructions (Human CCL‐5 (RANTES) and Human MCP‐1 Sandwich ELISA Kit, Proteintech, USA). Briefly, the enzyme immunoassay detected and quantified the protein levels of RANTES or MCP‐1 present in the samples. The test was performed on wells precoated with an antibody specific for human RANTES or MCP‐1. After washing with physiological solution, a biotinylated specific antibody was used to detect the bound RANTES or MCP‐1 proteins, and the signal was developed with streptavidin‐HRP, followed by tetramethylbenzidine (TMB) reagent. The development was halted with sulfuric acid, and the intensity of the color was measured at 450 nm with a correction wavelength set at 630 nm.

### 2.6. Study of Antiproliferative Activity

The antiproliferative activity of MFAT secretomes on cell proliferation was assessed using a cell viability MTT test (3‐(4,5‐dimethylthiazol‐2‐yl) 2,5‐diphenyltetrazolium bromide) after 7 days, as previously described [23, 24]. The test was performed on normal cells [mesenchymal stromal cells from adipose tissue (AT‐MSCs), hSDFs, keratinocytes (HaCaT), and tumor cells (human melanomas A375 and M20, human pancreatic adenocarcinoma CFPAC‐1)]. The results were expressed as a percentage of viability, normalized to untreated cells considered as 100%.

### 2.7. Statistical Analysis

Data obtained from three independent experiments were expressed as mean ± standard deviation (SD). Differences between mean values were evaluated using Analysis of Variance (ANOVA) with a Student‐Newman‐Keuls Multiple Comparisons Test performed by the GRAPHPADINSTAT program (GraphPad Software Inc., San Diego, CA, USA). *p*‐Values ≤ 0.05 were considered statistically significant. The linearity of response and correlation were analyzed using regression analysis with Excel 2013 software (Microsoft, Inc.).

## 3. Results

### 3.1. Expression of Adhesion Molecules

**Figure 1:**
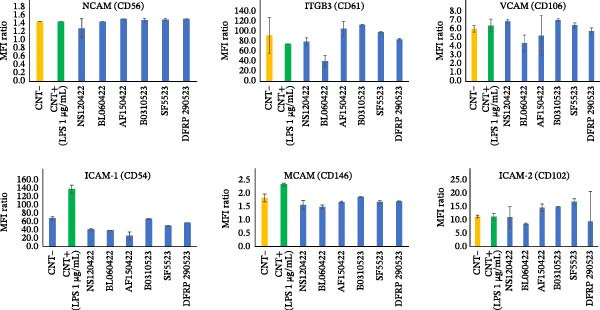
Cytofluorimetric analysis of adhesion molecules. The histograms show the expression data of the indicated adhesion molecules, represented as the ratio between the mean fluorescence intensity (MFI) of the specific antibody (NCAM, ITGB3, VCAM, ICAM‐1, MCAM, and ICAM‐2) and the MFI of the corresponding isotype control. MFI ratio values greater than 1 indicate the expression of the molecule on the cell surface. CNT− = negative control, CNT+ = positive control with lipopolysaccharide (LPS). Data are presented as the mean ± standard deviation of three independent replicates.

The expression of adhesion molecules on U‐937 cells was assessed by flow cytometry, as shown in Figure 1. The ratio between the mean fluorescence intensity (MFI) of the specific antibody and the MFI of the corresponding isotype control was calculated. An MFI ratio greater than 1 indicates the presence of the molecule on the cell surface Overall, the expression of NCAM, MCAM, VCAM, and ICAM‐2 was low (MFI ratio < 10), and no significant changes were observed in response to the secretomes from different donors. One secretome caused a slight inhibition of ITGB3 and VCAM, but this effect did not reach statistical significance (*p* > 0.05). Notably, ICAM‐1 expression was strongly inhibited by all secretomes.

As shown in Figure 2, LPS treatment significantly increased ICAM‐1 expression on macrophages (*p* < 0.05), with an MFI ratio of 137 ± 9.7, whereas treatment with all secretomes led to a significant 3‐fold decrease (*p* < 0.05) in ICAM‐1 expression, with an MFI ratio of 45.7 ± 2.3. A cytogram in Figure 2b further illustrates these findings, showing the distinct peaks of ICAM‐1 expression in the negative control, positive control (LPS treatment), and secretome‐treated groups. In conclusion, despite some variability between donors, all secretomes consistently inhibited ICAM‐1 expresson.

Figure 2ICAM‐1 expression. (a) The histogram reports ICAM‐1 expression, calculated as the ratio between the mean fluorescence intensity (MFI) of the specific antibody and that of the corresponding isotype control. ICAM‐1 expression is upregulated by LPS stimulation and markedly suppressed by all secretomes. The MFI ratio significantly increases after LPS treatment (*p* < 0.05) and is significantly reduced (*p* < 0.05) in the presence of each secretome. (b) Representative cytograms illustrating the gating strategy used to exclude dead cells, together with ICAM‐1 expression profiles and average values showing induction by LPS and inhibition by secretomes.

**Figure:**
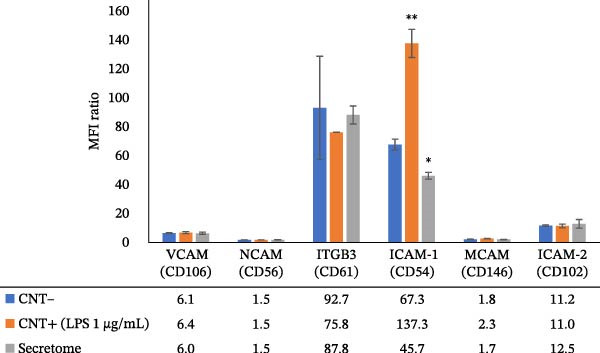
(a)

**Figure:**
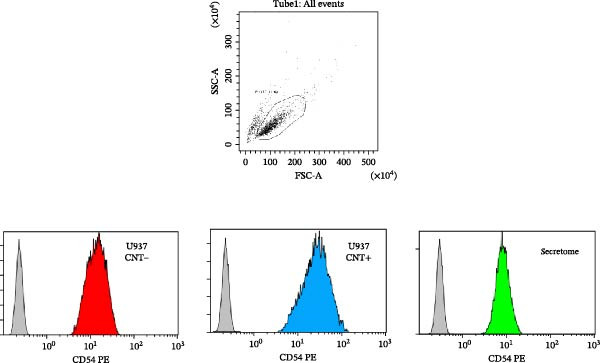
(b)

### 3.2. Expression of RANTES and MCP‐1

The production of RANTES and MCP‐1 was evaluated by ELISA, as shown in Figure 3. The levels of RANTES produced by U‐937 cells in the conditioned medium after stimulation with the secretomes are presented in Figure 3a. The histogram shows the means ± SD, highlighting significant stimulation by LPS (*p* < 0.01) and the varying effects of secretomes from different donors. Half of the secretomes did not stimulate RANTES production, while three samples induced a significant increase in RANTES secretion (*p* < 0.01). The mean RANTES production across all donor samples was 175.4 ± 14.23 pg/mL, which was not significantly different from that observed in unstimulated U‐937 cells (136.36 ± 19.90 pg/mL) but was significantly lower (*p* < 0.05) than the RANTES production in U‐937 cells stimulated with LPS that was about 1.7‐fold lower (230.25 ± 18.54 pg/mL).

Figure 3Evaluation of RANTES and MCP‐1 secretion. (a) The histogram shows the concentrations of RANTES (pg/mL) in the conditioned medium of U‐937 cells stimulated with the secretomes from various donors. LPS stimulation induces a significant increase in RANTES production (*p* < 0.01), and varying effects are observed with the secretomes from different donors. Three donor secretomes significantly stimulate RANTES production (*p* < 0.01). Data are presented as the mean ± standard deviation of three independent replicates. (b) The histogram shows the concentrations of MCP‐1 (pg/mL) in the conditioned medium of U‐937 cells stimulated with the secretomes of various donors. LPS stimulation results in a significant increase in MCP‐1 production (*p* < 0.05), while one secretome does not show a significant effect, with values similar to the unstimulated control (CTRL). Data are presented as the mean ± standard deviation of three independent replicates.

**Figure:**
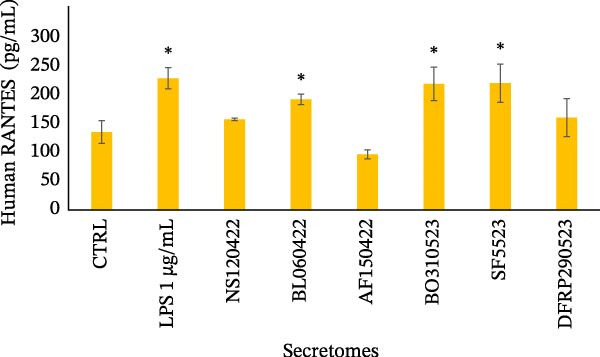
(a)

**Figure:**
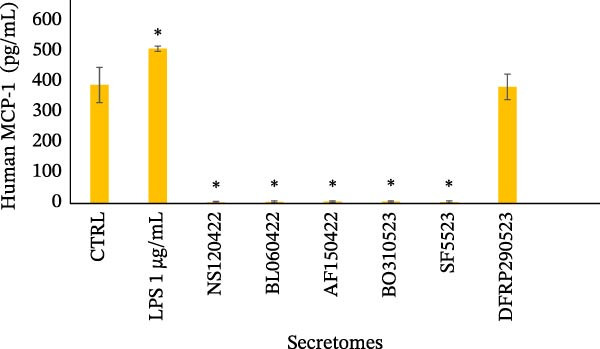
(b)

Figure 3b shows the concentrations of MCP‐1 produced by U‐937 cells when stimulated with the secretomes. The histogram illustrates a significant (*p* < 0.05) increase in MCP‐1 production when U‐937 cells were stimulated with LPS (510.26 ± 8.63 pg/mL). One secretome (384.24 ± 41.98 pg/mL) did not exert any significant effect, showing levels similar to those of the unstimulated control (390.49 ± 58.09 pg/mL). Notably, 83% of the donor secretomes strongly inhibited MCP‐1 production (*p* < 0.001) by the monocytes.

### 3.3. Activity on Cell Proliferation

We conducted preliminary investigations to assess whether the preparations had any significant impact on the proliferation of normal and tumor cells, which is an important factor for evaluating safety in clinical use. Although the results indicated varying effects depending on the donor’s preparation, most of the preparations did not significantly alter the proliferation of the studied cell lines. The sensitivity of normal cells to the same secretome preparation varied: AT‐MSCs showed greater sensitivity to the inhibitory effects compared to keratinocytes and fibroblasts, which were more resistant (Figure 4a). Additionally, while there were differences in the effects of the preparations from various donors on tumor cells, when considering all the data together, a trend of antitumor activity was observed rather than stimulation (Figure 4b).

Figure 4Effect of secretomes on normal and tumoral cell lines. (a) Inhibitory effect of secretomes on nontumoral cell proliferation. The effect on the proliferation of adipose tissue‐derived MSCs (AT‐MSCs), human stromal dermal fibroblasts (hSDFs), and human keratinocytes (HaCaT) is shown, expressed as μg/mL of secretome proteins. Proliferation was evaluated after 7 days using an MTT assay. Each point represents the mean ± standard error of the mean (SEM) from three replicates. (b) Inhibitory effect of secretomes on tumor cell proliferation. The effect on the proliferation of human pancreatic adenocarcinoma (CFPAC‐1) and human melanoma (M20 and A375) cell lines is shown, expressed as μg/mL of secretome proteins. Proliferation was evaluated after 7 days using an MTT assay. Each point represents the mean ± standard error of the mean (SEM) from three replicates.

**Figure:**
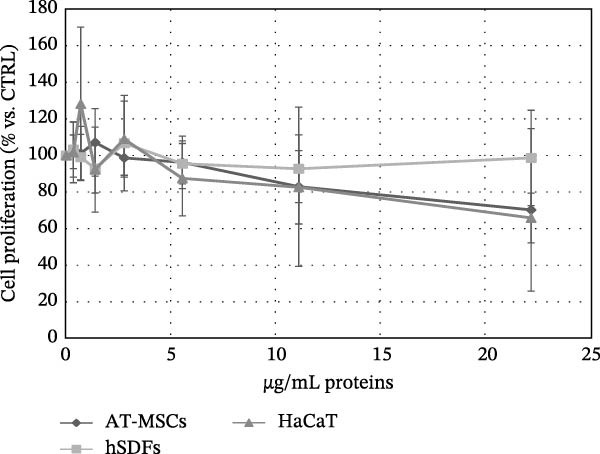
(a)

**Figure:**
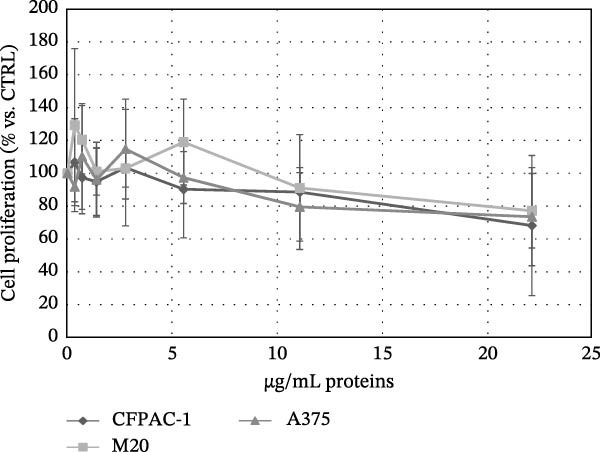
(b)

## 4. Discussion

In this study, we processed LA to obtain MFAT without using enzyme treatments, based on a procedure that has shown positive effects in patients with various pathologies [7, 25], particularly in osteoarthritis [4, 26]. The primary aim of this study was to investigate by in vitro studies whether the clinical benefits of MFAT could be associated with the anti‐inflammatory activity of its secretome. It is important to underline that the literature includes data supporting the benefits of Microfragmented Adipose Tissue (MFAT) treatment in both animals and humans. Moreover, several studies have investigated the effects of whole MFAT, focusing either on its cell/tissue‐based mechanisms or on its cell‐derived secretome [27]. Our study specifically explores the activity of tissue‐based secretomes—defined as the ensemble of bioactive molecules and extracellular vesicles, such as exosomes, released into a hydrophilic environment (i.e., a culture medium) by MFAT. This approach allows for the evaluation of MFAT‐derived factors independently of the cellular component, shedding light on their potential therapeutic role. To explore this, MFAT from different donors was cultured in serum‐free medium for 7 days to produce a conditioned medium (secretome), which was then tested on U937‐derived macrophage‐like cells, a widely used monocyte/macrophage cell line to study inflammation [9]. Previous studies from our group have demonstrated that MFAT maintains high cell viability and structural integrity during culture for at least 14 days, with only minimal reduction of MSC numbers observed after 28 days [28]. This stability is likely due to the intrinsic structural characteristics of MFAT, which is composed of small, homogeneous aggregates that naturally preserve their cellular content, acting as a “bioreactor” [29]. This evidence supports the reliability of the secretome obtained under our culture conditions. Upon stimulation with LPS, U‐937 cells undergo various cellular changes, including alterations in CAMs expression, although the extent of these changes can vary based on the activation pathway and cellular responses to LPS [30, 31]. As shown in Figure 1, the baseline expression of CAMs in U‐937 cells varied, as indicated by the MFI ratio. Notably, ITGB3, which is highly expressed in U‐937 cells, was not modulated by MFAT secretomes. ITGB3 plays a critical role in the movement of immune cells to sites of injury or infection, which is essential for pathogen defense. Despite its pro‐inflammatory roles, it can also exhibit anti‐inflammatory effects during the resolution of inflammation by promoting tissue repair, angiogenesis, and modulating platelet functions, making its role context‐dependent [32].

A key finding in our study was that LPS stimulation led to an increase in ICAM‐1 expression in U‐937 cells, which was significantly inhibited by all secretomes (*p* < 0.05). This is important because ICAM‐1 is an inflammatory molecule crucial for recruiting immune cells to sites of infection or injury and amplifying the inflammatory response. It also plays a role in chronic inflammation and tissue damage in various diseases [33]. While ICAM‐1 is traditionally considered pro‐inflammatory, it also has recognized anti‐inflammatory roles, contributing to the timely resolution of inflammation, wound healing, and macrophage polarization [13, 14, 30, 34–36]. In the tumor microenvironment, macrophage‐expressed ICAM‐1 is associated with a pro‐inflammatory phenotype. Figenschau et al. [37] noted that ICAM‐1 is expressed in aggressive breast cancer subtypes and can be induced by well‐known pro‐inflammatory cytokines. In our study, although we did not assess ICAM‐1 expression on tumor cells under secretome stimulation, we did examine the secretome’s impact on the proliferation of various tumor cell lines. The analysis revealed significant donor‐to‐donor variability, but overall, the data suggested that the secretome may exert antitumor activity rather than stimulation (Figure 4b). Several mechanisms could explain the LPS‐induced ICAM‐1 expression in U‐937 cells, with NF‐κB, MAPK, oxidative stress, and cytokine interactions being the most commonly implicated [38, 39]. The experimental conditions (such as the presence of other cytokines and donor variability in MFAT composition) could influence the outcome. It is well established that age, metabolic health, and obesity can significantly affect the biological activity of adipose tissue‐derived molecules. For example, adipose tissue from older individuals tends to release more inflammatory cytokines compared to that from younger individuals. For this study, MFAT was collected from healthy donors with an average age of 54 years. Since adipose tissue is an active endocrine tissue, it can release various molecules (adipokines, cytokines, chemokines, and oxidized lipids) in amounts that may influence inflammation mechanisms, even in healthy individuals. The most important observation from our study is that the inhibition of ICAM‐1 expression by the secretome was consistent across all donors, suggesting that adipose tissue contains bioactive molecules capable of modulating the signaling pathways responsible for ICAM‐1 induction. One potential mechanism for this effect could be the presence of adiponectin, a molecule known for its anti‐inflammatory properties, which may suppress ICAM‐1 expression by inhibiting pro‐inflammatory pathways like NF‐κB [40]. Other possible mechanisms could involve molecules such as IL‐10, negative regulators of NF‐κB, or microRNAs. In our preliminary lipidomic analysis of the MFAT secretome, we identified a significant release of oxidized triacylglycerols (oxTG) (unpublished data). While no studies have specifically investigated the relationship between oxidized TG and ICAM‐1 expression in U‐937 cells, the possibility that oxTG may interfere with signaling pathways cannot be ruled out. This interference could potentially lead to reduced ICAM‐1 expression by disrupting NF‐κB signaling or altering lipid rafts and membrane structures, thereby affecting receptor‐mediated signaling processes. We also observed that the MFAT secretomes strongly inhibited MCP‐1 chemokine production in U‐937 cells, with one donor’s secretome showing no modulation. MCP‐1 is an important chemokine that regulates the migration of inflammatory cells and has been implicated in the pathogenesis of various diseases, including COVID‐19 [41]. A variation in the ability of the secretomes to modulate RANTES production was also noted. Half of the secretomes stimulated RANTES production, suggesting that donor characteristics may influence this response. RANTES is a pro‐inflammatory cytokine that regulates immune cell recruitment to sites of inflammation [42]. Our study suggests that the MFAT secretome does not promote inflammation but instead appears to reduce certain monocyte inflammatory functions, particularly by downregulating ICAM‐1 expression. These findings suggest that the paracrine activity of MFAT may play a significant role in modulating inflammation, potentially accelerating recovery in osteoarthritis patients. Given that the secretome contains active molecules involved in inflammatory processes, its use as fresh MFAT in clinical settings may be beneficial [1]. Further investigation is needed to explore the role of lipids and molecules involved in immune cell recruitment during inflammation in the osteoarthritic microenvironment [43]. Additionally, the secretomes did not enhance cell proliferation but instead seemed to inhibit tumor cell growth, which may indicate its safety for use in clinical trials, especially in osteoarthritis treatment [44, 45]. Although conducted on a limited number of donors, our findings provide preliminary evidence that MFAT‐derived factors do not exert a proliferative stimulus, thereby not suggesting any potential safety concern in this regard. Nevertheless, the study remains exploratory and is inherently constrained by the small cohort size and the inter‐donor variability observed. Moreover, the inflammatory response is an inherently complex and heterogeneous process, which requires further investigation using alternative models beyond U937 cells. Indeed, while U937 cells provide a reproducible and well‐characterized monocyte/macrophage system for preliminary screening, they cannot fully reproduce the complexity of immune interactions in vivo; therefore, validation in primary human cells or more complex models will be essential. To this end, our data could also be validated in primary human macrophages or other monocyte/macrophage models to broaden, deepen, and generalize the clinical relevance of our observations.

Nevertheless, our findings support and reinforce several clinical observations suggesting an anti‐inflammatory effect of MFAT. Although endotoxin contamination must always be considered, MFAT is a clinically validated product routinely applied intraoperatively, and in our study the functional response of U937 cells showed no pro‐inflammatory activation. Together, these observations support the safety of MFAT and suggest that endotoxin contamination did not affect our experimental results.

Another aspect to consider is the presence of joint inflammation that may influence MFAT efficacy [46], potentially because excessive inflammation could exceed its modulatory capacity. This observation further supports the relevance of anti‐inflammatory mechanisms in MFAT’s clinical action. Expanding the sample size in future studies would be valuable to determine whether distinct donor subpopulations exist with different capacities to stimulate RANTES production. It is also important to note that secretome preparation involves minimal manipulation, potentially simplifying clinical application by avoiding the stringent requirements of cGMP protocols and reducing the costs associated with cell‐based therapies. Furthermore, if validated by in vivo studies, the use of secretome could address some of the technical limitations of MFAT—particularly its current restriction to local treatments—by enabling systemic administration.

## Author Contributions

Conceptualization and study design were carried out by Augusto Pessina, Francesca Paino, and Giulio Alessandri. Material preparation and data collection were performed by Valentina Coccè, Eleonora Martegani, Barbara Manfredi, and Emilio Ciusani. Data analysis and interpretation were conducted by Luisa Doneda, Emilio Ciusani, and Valentina Coccè. The manuscript was drafted and critically revised by Augusto Pessina, Francesca Paino, and Giulio Alessandri. Elena Colombani, Carlo Tremolada, and Bruno Giannì provided resources support for the project.

## Funding

The authors declare that no funds, grants, or other support were received during the preparation of this manuscript. Open access publishing facilitated by Universita degli Studi di Milano, as part of the Wiley ‐ CRUI‐CARE agreement.

## Disclosure

All authors read and approved the final manuscript.

## Ethics Statement

The study was conducted according to the guidelines of the Declaration of Helsinki and approved by the Research Ethics Committee of the University of Milan. Approval Number: n.58/23, C.E.UNIMI, date of approval 25.05.23.

## Consent

Informed consent was obtained from all subjects involved in the study.

## Conflicts of Interest

The authors declare no conflicts of interest.

## Data Availability

The data that support the findings of this study are available from the corresponding author upon reasonable request.
